# Extracellular vesicle-derived microRNAs in renal cell carcinoma: biological roles and clinical applications

**DOI:** 10.3389/fcell.2025.1694257

**Published:** 2025-10-21

**Authors:** Jiahui He, Lanfeng Wang, Bingqing Yu, Wengbin Huang, Chen Ouyang, Mingcan Zhou, Rong Hu, Zhiping Chen

**Affiliations:** ^1^ Department of Laboratory Medicine, First Affiliated Hospital of Gannan Medical University, Ganzhou, Jiangxi, China; ^2^ College of Medical Technology, Gannan Medical University, Ganzhou, Jiangxi, China; ^3^ Department of Nephrology, First Affiliated Hospital of Gannan Medical University, Ganzhou, Jiangxi, China; ^4^ First Clinical Medical College, Gannan Medical University, Ganzhou, Jiangxi, China

**Keywords:** extracellular vesicles, miRNAs, renal cell carcinoma, kidney cancer, biomarker

## Abstract

Renal cell carcinoma (RCC) is a common malignant tumour of the urinary system, characterised by high heterogeneity and a tendency to metastasise, with poor prognosis in advanced patients. Although surgical resection and targeted therapies such as tyrosine kinase inhibitors and immune checkpoint inhibitors have significantly improved survival outcomes in some patients, drug resistance and recurrence remain clinical challenges. In recent years, extracellular vesicles (EVs) and the microRNAs (miRNAs) they carry have emerged as a research hotspot due to their critical roles in tumour initiation, progression, immune regulation, and drug resistance. This systematic review summarises the biological functions of EVs-derived miRNAs in renal cell carcinoma and their potential applications in clinical diagnosis and treatment, with a focus on their value in diagnosis, prognosis, immune regulation, and prediction of treatment response.

## 1 Introduction

Renal cell carcinoma (RCC) is one of the most common malignant tumours of the urinary system, with over 400,000 new cases diagnosed globally each year, making it one of the leading causes of cancer-related deaths. The incidence of RCC is on the rise in both developed and developing countries (Chen et al., 2023; Sun et al., 2023; Grünwald et al., 2024). Based on histological characteristics, the most common subtypes of RCC are clear cell type, papillary type (Type I and Type II), and chromophobe type, accounting for 70%–90%, 10%–15%, and 3%–5% of all renal malignant tumours, respectively. RCC accounts for 2%–3% of all cancers and is the most lethal urogenital system cancer, with a mortality rate of 30%–40%, compared to approximately 20% for bladder cancer and prostate cancer. The incidence of RCC continues to rise, varying globally with higher rates in developed countries than in developing countries (Stepanovska Tanturovska et al., 2023). Despite recent advances in surgical techniques and the introduction of novel targeted therapies such as tyrosine kinase inhibitors (TKIs) and immune checkpoint inhibitors (ICIs), the prognosis for patients with advanced RCC remains poor, with a five-year survival rate below 15%. The primary challenges in current treatment include the presence of drug resistance mechanisms and the lack of reliable early diagnostic and prognostic biomarkers (Chen et al., 2020; Motzer et al., 2022; Delcuratolo et al., 2023).

**FIGURE 1:**
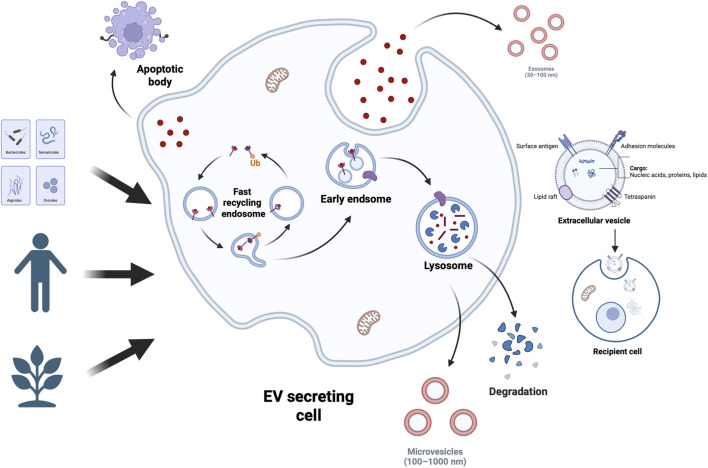
EV Types and Sources. Extracellular vesicles (EVs) encapsulate bioactive components like proteins, nucleic acids, and lipids—lipids act as structural elements and reflect cellular traits. Cells across tissues use EVs for intercellular communication, releasing them into body fluids. A large share of human EVs comes from stem cells; EVs are also actively produced and released by organisms from plants to bacteria.

Extracellular vesicles (EVs) have been demonstrated to serve as crucial intercellular communication mediators within the tumour microenvironment (TME) (Kalluri and LeBleu, 2020; Urabe et al., 2020; Xiao Y. et al., 2020; Huang et al., 2021; Marar et al., 2021; Fusco et al., 2024). Enclosing diverse bioactive components including proteins, lipids, and nucleic acids, EVs modulate recipient cell behaviour, thereby influencing numerous physiological and pathological processes such as TME formation and evolution (Maacha et al., 2019; Ortiz, 2021; Kita and Shimomura, 2022; Kumar et al., 2024; Rai et al., 2024). The mechanisms of EV-mediated cellular communication are diverse, encompassing activation of membrane surface receptors, Messenger RNA (mRNA) transport and translation, transfer of microRNA (miRNAs) and target mRNAs, delivery of functional proteins, and activation of signalling pathways via internalisation (D'Souza-Schorey and Clancy, 2012; Svensson and Belting, 2013; Maeda et al., 2023). Among these, miRNAs have emerged as a current research focus due to their crucial role in gene expression regulation and their extensive involvement in tumour progression, metastasis, angiogenesis, and immunomodulation. Valadi et al. were the first to demonstrate that miRNAs can be transported between cells via extracellular vesicles while retaining biological activity in recipient cells (Valadi et al., 2007). miRNA constitutes one of the most abundant RNA components within EVs, accounting for up to 40% of RNA in human plasma EVs as revealed by RNA sequencing (Mittelbrunn et al., 2011; Makarova et al., 2021; Yang et al., 2021; Xiong et al., 2023). Studies have shown that miRNAs are predominantly enclosed within extracellular vesicles (EVs), where vesicular encapsulation protects them from nuclease degradation in body fluids, thereby facilitating intercellular communication (Kogure et al., 2019). EVs are widely distributed across various biological fluids, including plasma, saliva, urine, milk, and cerebrospinal fluid. Notably, the abundance of miRNAs carried by EVs from milk and cerebrospinal fluid may exceed that of plasma-derived EVs (Tabatabai et al., 2025). Whether derived from normal or tumour tissues, EVs serve as carriers for both miRNA and pathogenic proteins, potentially contributing to the onset and progression of certain diseases (Saeedi et al., 2021; Doncheva et al., 2022; Kumar et al., 2024) (Figure 1).

microRNAs (miRNAs) are a class of small non-coding RNAs that primarily suppress gene translation by targeting messenger RNAs (mRNAs), and participate in diverse biological processes including cell differentiation, proliferation, apoptosis, and development (Lu et al., 2005; Filella and Foj, 2017; Selvaskandan et al., 2023). In malignant tumours, miRNAs may function as either tumour suppressors or oncogenes, exhibiting characteristic alterations in their expression levels (Guil and Esteller, 2009; Hill and Tran, 2021; Li B. et al., 2021). The differential expression of miRNAs between normal and cancerous cells renders them ideal candidate molecules for tumour biomarkers (Chow et al., 2010). miRNAs within tumour-derived EVs can further enhance the invasive and metastatic capabilities of tumour cells by modulating the tumour microenvironment (TME) (Nishida-Aoki and Ochiya, 2015; Tkach and Théry, 2016).

Recent studies have revealed significant differences in the expression of EVs-miRNA between healthy individuals and RCC patients (Nawaz et al., 2014), suggesting broad application prospects in non-invasive diagnosis and prognostic assessment. Furthermore, engineered miRNA delivery systems based on EVs have demonstrated potential therapeutic value (Rädler et al., 2023). The high stability of miRNAs in bodily fluids such as plasma and urine render them ideal molecules for liquid biopsy. Nevertheless, technical challenges persist in clinical implementation, including standardization of isolation methods, target specificity, and potential off-target effects. This paper aims to provide a systematic review of research progress on EVs-miRNA in RCC, focusing on their role in tumour biology, potential as diagnostic biomarkers, and therapeutic applications. It also explores challenges and future directions in their clinical translation.

## 2 The biological basis of EVs and miRNAs

Extracellular vesicles are a class of membrane-bound nanovesicles actively secreted by cells, carrying diverse biomolecules such as proteins and nucleic acids, particularly microRNA (miRNA), which play crucial roles in tumourigenesis and progression. These vesicles can be readily extracted from peripheral blood and other bodily fluids, rendering them potential tools for non-invasive tumour diagnosis (Raposo and Stoorvogel, 2013; Kogure et al., 2019; Munir et al., 2020). The biological effects of extracellular vesicles on surrounding or distant target cells are primarily determined by their biomolecular cargo. Their lipid composition includes sphingolipids, cholesterol, phosphatidylserine, saturated fatty acids, and sphingosine—substances also prevalent in the plasma membrane (Trajkovic et al., 2008; Skotland et al., 2020). Research indicates that sphingosine plays a direct role in the formation of internalised vesicles (ILVs) within the lumen of multivesicular bodies (MVBs). Inhibition of neutral sphingomyelinase significantly reduces extracellular vesicle release, further validating this lipid’s critical function in extracellular vesicle biogenesis (Menck et al., 2017).

The proteome of extracellular vesicles primarily comprises proteins involved in membrane transport, such as tetramembrane proteins (CD63, CD81, CD82, and CD9), whose recruitment depends on the ALIX and ESCRT-III pathways (Larios et al., 2020). Furthermore, extracellular vesicles are rich in heat shock proteins (Hsp60, Hsp70, Hsp90), integrins, and class II major histocompatibility complex (MHC II) molecules (Clayton et al., 2005). Notably, extracellular vesicles do not simply represent the protein expression profile of the parent cell; rather, they selectively enrich certain proteins through specific mechanisms. Among these, ubiquitination is recognised as a key regulatory step mediating protein binding to the ESCRT complex and facilitating their incorporation into vesicles (Larios et al., 2020).

miRNAs are regulated both by their biogenesis mechanisms and by the sequence characteristics of the miRNAs themselves. Although the ESCRT system plays a central role in the formation of multi-vesicular bodies (MVBs) and EV release, studies indicate that knocking down key ESCRT proteins does not affect miRNA content (Kosaka et al., 2010). Interestingly, while knocking down the ESCRT-III-associated protein Alix does not affect total extracellular vesicle release, it leads to a significant reduction in miRNA content (Iavello et al., 2016).

Certain miRNA sequences contain conserved domains known as EXO-motifs, which bind to specific RNA-binding proteins such as hnRNPA2B1 and SYNCRIP, thereby facilitating their incorporation into extracellular vesicles (Santangelo et al., 2016). Previous studies have identified short motif sequences (e.g., GGAG in miR-198 and UGCA in miR-601) that regulate miRNA loading into extracellular vesicles, with point mutations altering these motifs significantly impacting miRNA efficiency (Villarroya-Beltri et al., 2013). Recent studies have further revealed an association between AGO2 and extracellular vesicle-associated miRNAs, with AGO2 being identified in extracellular vesicle proteomics via mass spectrometry (MS) or Western blotting analyses (Goldie et al., 2014; Zhang J. et al., 2015). Knockout of AGO2 was found to reduce the types or abundance of preferentially exported miRNAs in extracellular vesicles derived from HEK293T cells (Guduric-Fuchs et al., 2012). Further investigations indicate that YBX1 (Y-box protein I) also binds to miR-223 and miR-144, regulating their packaging into vesicles (Ung et al., 2014; Shurtleff et al., 2016). Collectively, these findings suggest that specific sequences within miRNAs may guide their incorporation into extracellular vesicles.

Extensive research indicates that miRNAs within extracellular vesicles participate in the formation of multiple tumour types and serve as sensitive biomarkers for cancer diagnosis (Aguilar-Hernandez et al., 2021; Lu et al., 2021; Romano et al., 2021; Maruoka et al., 2022; Wang et al., 2022; Genova et al., 2024; Kural et al., 2024). Compared to free-floating miRNAs, those derived from extracellular vesicles exhibit enhanced stability, owing to the protective double-layered membrane structure that shields them from degradation by endogenous nucleases or phagocytes such as macrophages. The nanoscale dimensions and membrane protective mechanisms of extracellular vesicles prolong the half-life of miRNAs in bodily fluids, enhancing their biological functionality (Sun et al., 2022). Upon release into bodily fluids, extracellular vesicles may be internalised by neighbouring or distant cells, where the encapsulated miRNAs can regulate a range of biological processes including immune evasion, tumour microenvironment modulation, angiogenesis, metastasis, and drug resistance development (Sun et al., 2018). Consequently, the role of EVs-miRNAs in cancer progression has garnered significant attention. For instance, Fabbri (2012) demonstrated that miRNAs within lung cancer-derived extracellular vesicles promote tumour cell proliferation and metastasis by activating Toll-like receptors (TLRs). Zhou et al. (2014) further reported that extracellular vesicles secreted by tumour cells carrying miR-105 disrupt tight junctions between vascular endothelial cells, compromising the natural barrier and thereby promoting metastasis. Additionally, studies on renal cell carcinoma (RCC) have identified circulating EVs enriched with multiple miRNAs associated with metastasis and invasion, including miR-200c, miR-92, miR-141,miR-19b, miR-29a, miR-29c, miR-650, and miR-151 (Chow et al., 2010; Grange et al., 2011). These miRNAs hold promise as biomarkers for renal carcinoma progression, demonstrating significant research and clinical application value.

## 3 The function of EVs carrying miRNAs in renal cell carcinoma

### 3.1 Tumorigenesis and progression

In recent years, multiple studies have progressively revealed the pivotal regulatory role of miRNAs carried by EVs in the development and progression of clear cell renal cell carcinoma (ccRCC). DISA et al. discovered that PTEN serves as a common target gene for hsa-miR-301a-3p, hsa-miR-200c-3p, and hsa-miR-25-3p. Studies indicate that these miRNAs are significantly enriched in plasma extracellular vesicles during the presence of primary tumours, with their expression levels markedly decreasing following tumour resection. This suggests that EVs-miRNAs may regulate tumour progression at the post-transcriptional level by activating the PI3K/AKT signalling pathway (Dias et al., 2020). Notably, hsa-mir-301a-3p exhibited a declining trend post-tumour resection, with its levels continuing to decrease during follow-up. However, its expression significantly increased in the metastasis cohort, suggesting this miRNA may play a crucial role in metastasis and holds potential as a prognostic biomarker. In contrast, the EV-derived levels of hsa-miR-1293 progressively increased after tumour resection until follow-up yet were markedly reduced in the metastatic cohort. This indicates the miRNA’s potential tumour-suppressing function and its possibility as a biomarker for metastatic disease in ccRCC patients (Dias et al., 2020). Among these, hsa-miR-301a-3p exhibited a consistent decrease in expression following surgery but was significantly elevated in patients with metastasis during follow-up, indicating its potential involvement in metastatic dissemination and its utility as a prognostic biomarker. Conversely, hsa-miR-1293 displayed increased EV expression post-surgery but was significantly downregulated in metastatic cases, suggesting its potential tumor-suppressive role and prognostic relevance in identifying metastatic ccRCC (Dias et al., 2020). Ding et al. using small RNA sequencing, demonstrated that miR-181d-5p was highly enriched in EVs derived from cancer-associated fibroblasts (CAFs) in RCC patients. This miRNA directly suppressed RNF43 expression in RCC cells and activated the Wnt/β-catenin pathway, thereby enhancing cancer stemness and promoting tumor progression (Ding et al., 2022). Additionally, RAB27 A/B has been shown to regulate EV-miRNA secretion. Decreased RAB27A expression was closely associated with lymph node metastasis and poor prognosis in RCC (Chen et al., 2012; An et al., 2019). Song et al. further reported that elevated RAB27A expression promoted RCC cell secretion of miR-127-3p, which, through EV-mediated transfer, upregulated MYCN expression and enhanced tumor invasiveness (Song et al., 2024). miR-9-5p has also been implicated in RCC progression. Song et al. found that it was significantly elevated in serum from advanced RCC patients and positively correlated with TNM stage and Fuhrman grade. Functional assays demonstrated that EV-derived miR-9-5p promoted proliferation and invasion of A-704 cells by downregulating SOCS4, both *in vitro* and *in vivo*, supporting its role as a diagnostic and prognostic biomarker (Song et al., 2020).

Further *in vitro* and *in vivo* studies confirmed that ccRCC-derived EVs transmit miR-27a, which suppresses its target gene SFRP1 while enhancing vascular endothelial growth factor (VEGF) and tumour necrosis factor-α (TNF-α) expression, thereby promoting RCC cell viability, migration, and angiogenesis (Hou et al., 2021). Xuan et al. observed significantly downregulated miR-549a expression in TKI-resistant ccRCC cells and their extracellular vesicles. Extracellular vesicle-derived miR-549a inhibits tumour angiogenesis and reduces endothelial cell migration by binding to the 3′-UTR region of HIF-1α, thereby suppressing its expression (Xuan et al., 2021).

Li et al. further observed that miR-15a, upregulated in EVs, enhances the epithelial-mesenchymal transition (EMT) capacity of ccRCC cells by downregulating BTG2 and activating the PI3K/AKT pathway (Li D. Y. et al., 2021). Furthermore, Wang et al. observed that in metastatic ccRCC patients, cancer stem cell (CSC) extracellular vesicles induce EMT by transporting miR-19b-3p to tumour cells and suppressing PTEN gene expression. CD103+-guided CSC extracellular vesicles target cancer cells and organs, conferring enhanced lung metastatic potential to ccRCC. Consequently, CD103+ extracellular vesicles also emerge as a potential metastatic diagnostic biomarker (Wang L. et al., 2019).

### 3.2 Immune regulation

Tumour-associated macrophages (TAMs) play a pivotal role in regulating the tumour microenvironment (TME) and promoting tumour initiation and progression (Pollard, 2004). Increasing evidence indicates that TAMs are not only one of the predominant cell types within the TME, but also participate in the complex processes of cancer through multiple mechanisms including immune suppression, promotion of tumour progression, metastasis, and drug resistance (Pathria et al., 2019; Wei et al., 2019). Macrophages can be categorised into pro-inflammatory M1 and immunosuppressive M2 types based on their functional state. TAMs exhibit phenotypic and functional characteristics closer to M2 macrophages, which are strongly associated with tumour-promoting properties (Boutilier and Elsawa, 2021). Multiple studies demonstrate that TAM infiltration correlates closely with poor prognosis across various cancer types, including RCC (Komohara et al., 2011; Zhou et al., 2015; Chen et al., 2017). Consequently, targeting TAMs presents a potential therapeutic strategy that may offer novel insights into the interactions between the tumour microenvironment and tumour cells (Pyonteck et al., 2013; Ries et al., 2014). In RCC, TAMs have been demonstrated to promote tumour cell migration and tumour growth (Kadomoto et al., 2019; Schnetz et al., 2020).

Regarding specific mechanisms, research has revealed that upregulation of HIF-1α in macrophages promotes miR-193a-5p expression. This microRNA is subsequently transported to RCC cells via extracellular vesicles, targeting the 3′-untranslated region (3′-UTR) of TIMP2 mRNA. This downregulates TIMP2 expression, thereby enhancing tumour angiogenesis and invasive capacity. Inhibition of miR-193a-5p in extracellular vesicles derived from TAMs has been shown to significantly attenuate RCC progression and metastasis, offering a novel therapeutic approach targeting TAM-associated miRNAs (Liu et al., 2022). In a ccRCC model, Feng et al. discovered that extracellular vesicles derived from M2-polarised macrophages transport miR-342-3p to target and inhibit NEDD4L, thereby blocking the ubiquitination and degradation of CEP55 and activating the PI3K/AKT/mTOR pathway. This ultimately enhances the proliferation, migration, and invasive capacity of RCC cells. This research offers fresh perspectives for developing therapeutic targets in RCC (Feng et al., 2021). Zhang et al. further indicated that extracellular vesicles derived from M2 macrophages serve as key mediators enhancing RCC cell migration and invasive potential. The miR-21-5p they enrich promotes distant tumour metastasis by downregulating PTEN expression and activating the Akt signalling pathway. *In vitro* and in xenograft models, this miRNA consistently demonstrated metastatic-promoting capabilities. Notably, application of miR-21-5p inhibitors reversed these pro-metastatic effects, opening novel pathways for intervening in TAM-mediated metastatic behaviour and offering a novel therapeutic strategy for preventing RCC metastasis (Zhang Z. et al., 2022). Furthermore, research indicates that extracellular vesicles derived from ccRCC cells can transport long non-coding RNA (lncARSR), activating the miR-34/miR-449-STAT3 signalling pathway. This induces the transformation of M1 macrophages towards an M2 phenotype, enhancing their phagocytic activity and promoting angiogenesis, thereby accelerating tumour progression (Zhang W. et al., 2022).

### 3.3 Drug resistance

With the widespread application of multi-targeted kinase inhibitors in the treatment of advanced ccRCC, the issue of drug resistance has progressively become a key obstacle affecting therapeutic efficacy. Sorafenib is a commonly used oral multi-targeted tyrosine kinase inhibitor in clinical practice. Its mechanism of action primarily involves inhibiting multiple angiogenesis and tumour proliferation-related signalling pathways, including: VEGFR-2, VEGFR-3, platelet-derived growth factor receptor-β (PDGFR-β), RAF-1,c-Kit, and FMS-like tyrosine kinase 3 (Flt-3) (Yu et al., 2015).

Research by He et al. revealed that tumour-derived extracellular vesicles can promote sorafenib resistance by transporting miR-31-5p. They further demonstrated that this microRNA targets the 3′-UTR of the MLH1 gene, leading to its downregulation and thereby inducing a sorafenib-resistant response (Yu et al., 2015). Additional studies have reported that miR-31-5p-enriched extracellular vesicles in ccRCC models mediate resistance signals by directly targeting the DNA mismatch repair-associated gene MutL homolog 1 (MLH1), thereby enhancing the sorafenib-resistant phenotype. Consequently, both miR-31-5p and its target genes may serve as predictive biomarkers and therapeutic targets for sorafenib resistance (He et al., 2020). Qu et al. further discovered that extracellular vesicles secreted by RCC cells deliver IncARSR, which promotes sunitinib resistance by competitively binding miR-34/miR-449, thereby enhancing AXL and c-MET expression in RCC (Qu et al., 2016).

**FIGURE 2:**
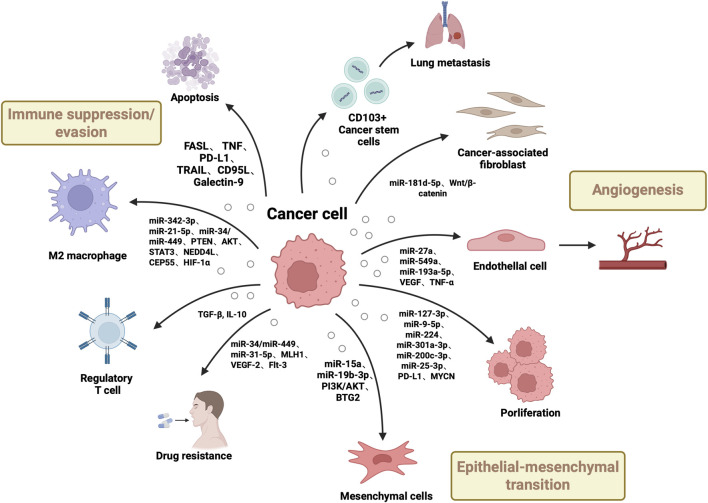
Roles of Renal Cell Carcinoma-Derived Extracellular Vesicles (EVs) in Tumorigenesis. This figure shows renal carcinoma EVs drive tumor development via multiple pathways: delivering growth-promoting miRNAs/oncogenes to boost cancer cell proliferation/dissemination, transferring molecules to induce epithelial-mesenchymal transition, conveying pro-angiogenic factors for neovascularization, and transporting immunosuppressive molecules to enable immune evasion, while interacting with stromal cells to remodel the tumor microenvironment.

In summary, EV-carried miRNAs exert core regulatory roles in RCC initiation, progression, immune modulation, and drug resistance mechanisms. By targeting multiple signalling pathways (e.g., PI3K/AKT, Wnt/β-catenin, STAT3), they influence cancer cell proliferation, migration, invasion, and metastatic nodule formation. Certain miRNAs (e.g., miR-301a-3p, miR-21-5p,miR-342-3p) are closely associated with prognosis, whilst miR-193a-5p, miR-9-5p, and miR-127-3p play crucial roles in immune regulation and invasive metastasis mediated by tumour-associated macrophages (TAMs) and cancer stem cells (CSCs). Furthermore, miR-31-5p has been demonstrated to contribute to sorafenib resistance by downregulating MLH1 expression, suggesting its potential for predicting and overcoming targeted therapy resistance. As the functional role of EV-associated miRNAs is increasingly elucidated, their clinical value as non-invasive biomarkers and therapeutic intervention targets becomes increasingly evident, offering broad prospects for the early diagnosis, dynamic monitoring, and personalised treatment of renal cell carcinoma (Figure 2, Table 1).

**TABLE 1:** Functions of EV-carried miRNAs in RCC.

Sample source	Number of samples	Dysregulated miRNAs	Physiological effect	Target genes	miRNA profiling	REF
Plasma-derived Evs	32 ccRCC patients with localized disease (before and after surgery) and in 37 patients with metastatic disease	miR-301a-3p↑miR-1293↓	miR-301a-3p activates PI3K/AKT to promote progression and metastasismiR-1293 acts as a tumor suppressor	PTEN	Small RNA-seq, RT-qPCR	Dias et al. (2020)
Cell-derived Evs	3 RCC patient pairs (tumor tissues: CAFs/adjacent normal tissues: NFs)	miR-181d-5p↑	Promotes tumor cell proliferation, migration, and invasion	RNF43	Small RNA-seq, qRT-PCR	Ding et al. (2022)
Cell-derived Evs	N/A	miR-127-3p↑	Promotes tumor cell migration, invasion, and metastasis and is associated with poor prognosis	MYCN	RT-qPCR、Next-Generation Sequencing	Song et al. (2024)
Serum-derived EVs	35 ccRCC patients (high expression)/31 ccRCC patients (low expression)	miR-9-5p↑	Promotes tumor cell proliferation and migration, positively associated with TNM stage and Fuhrman grade	SOCS4	qRT-PCR	Song et al. (2020)
Cell-derived Evs	N/A	miR-549a↓	Enhances vascular endothelial permeability and angiogenesis and promotes lung metastasis formation	HIF-1α	RT-qPCR, Small RNA-seq	Xuan et al. (2021)
Tissue-derived Evs	53 paired ccRCC tissues and adjacent normal tissues (31 stage I–II/22 stage III–IV patients)	miR-15a↑	Promotes tumor cell proliferation, migration, invasion, and EMT and aggravates ccRCC progression via PI3K/AKT activation	BTG2	Microarray, RT-qPCR	Li et al. (2021b)
Tissue-derived Evs	133 CCRCC patients (stage I–II, non-metastatic)/76 CCRCC patients (stage III–IV, metastatic)	miR-19b-3p↑	Enhances tumor cell migration, invasion, and metastasis and CD103⁺ exosomes preferentially target lung tissue to promote metastasis	PTEN	RT-qPCR, Small RNA-seq	Wang et al. (2019a)
Tissue-derived Evs	51 histologically confirmed ccRCC tissue samples (including 23 paired adjacent non-cancerous tissues)	miR-193a-5p↑	Enhances tumor cell migration and angiogenesis and promotes tumor progression and metastasis	TIMP2	RT-qPCR	Liu et al. (2022)
Cell-derived Evs	N/A	miR-342-3p↑	Promotes tumor cell proliferation, migration, invasion, and metastasis by inhibiting NEDD4L and stabilizing CEP55 to activate PI3K/AKT/mTOR signaling	NEDD4L, CEP55	RT-qPCR	Feng et al. (2021)
Cell-derived Evs	N/A	miR-21-5p↑	Promotes tumor cell proliferation, migration, invasion, and distant metastasis by downregulating PTEN and activating Akt signaling to induce EMT.	PTEN	RT-qPCR	Zhang et al. (2022b)
Cell-derived Evs	N/A	lncARSR↑→miR-34/miR-449	Induces macrophage polarization from M1 to M2 and promotes phagocytosis, angiogenesis, and tumor progression	STAT3	RT-qPCR	Zhang et al. (2022a)
Plasma-derived Evs	40 metastatic RCC patients (sorafenib treatment)	miR-31-5p↑	Promotes sorafenib resistance and enhances tumor cell proliferation and survival under drug pressure	MLH1	RT-qPCR, TaqMan miRNA probes	He et al. (2020)

## 4 EVs-miRNA serves as a biomarker for RCC

In recent years, liquid biopsy has garnered significant attention in both clinical practice and fundamental research as a non-invasive tool for tumour detection. Particularly in the early diagnosis of renal tumours, accurately distinguishing between benign and malignant small renal masses (SRMs) is crucial for determining the need for surgical intervention. Although the diagnostic accuracy of tissue biopsy continues to improve (Marconi et al., 2016), its invasive nature and procedural limitations remain obstacles to widespread clinical adoption. In contrast, miRNAs carried by EVs possess inherent protective mechanisms due to their membrane-enclosed structure, exhibiting enhanced stability and detection reproducibility in biological fluids such as plasma, serum, and urine. This offers promising clinical application prospects. Consequently, liquid biopsy strategies based on EVs-miRNA hold potential as a safer, more sensitive, and reproducible diagnostic approach for renal cancer. Simultaneously, identifying ccRCC patients at high metastatic risk is crucial for developing personalised monitoring plans, optimising adjuvant treatment decisions, and enabling early intervention against metastatic lesions. However, clinically validated molecular biomarkers for risk stratification in this patient cohort remain scarce. Notably, despite advances in targeted therapies such as TKIs and ICIs, predictive indicators for treatment efficacy remain limited (Linxweiler and Junker, 2020). Consequently, exploring EVs-miRNAs with high stability, specificity, and reproducibility as liquid biopsy biomarkers has become a core research direction for precision diagnosis and treatment of RCC. Previous studies have demonstrated that EV-associated miRNAs remain stable without degradation in preservation solutions under hypothermic ischemia conditions (4 °C) during liver transplantation (Vidal-Correoso et al., 2024). Ma et al. further showed that glycosylated extracellular vesicles preserve their miRNA cargo without degradation under storage at −80 °C and remain stable for up to 7 days at 4 °C (Ma et al., 2023). In addition, Muth et al. evaluated the effects of freeze–thaw cycles and room temperature incubation on plasma EV-miRNAs, and found that appropriate pre-analytical processing (e.g., platelet removal) markedly improves the stability and accuracy of EV-miRNA analysis (Muth et al., 2018). Collectively, these findings suggest that EV-associated miRNAs are feasible candidates for use as routine biomarkers. Numerous studies have reported that miRNAs in extracellular vesicles demonstrate significant potential in RCC diagnosis, prognostic assessment, and treatment response prediction (Butz et al., 2016; De Palma et al., 2016; Zhang et al., 2018; Song et al., 2019; Wang X. et al., 2019).

miR-210 and miR-1233, isolated from serum extracellular vesicles, have been validated as diagnostic biomarkers for clear cell renal cell carcinoma (ccRCC), exhibiting sensitivities of 70% and 81%, respectively, alongside specificities of 62.2% and 76.0%. Notably, these biomarkers demonstrate a significant decline following nephrectomy. Furthermore, Wang and colleagues reported that miR-210 could identify RCC with 82.5% sensitivity and 80.0% specificity. In this context, higher miR-210 levels were observed in more advanced cases and those with higher Fuhrman grading, independent of gender or age (Zhang et al., 2018; Wang X. et al., 2019). Fujii et al. further demonstrated that high expression of Exo-miR-224 correlates strongly with poor prognosis in ccRCC patients, manifesting as reduced survival times and accelerated tumour progression. Compared to the low-expression group, the high-expression Exo-miR-224 group exhibited significantly shorter progression-free survival, cancer-specific survival, and overall survival. In multivariate analysis, elevated Exo-miR-224 levels emerged as a significant prognostic risk factor across all studies. Co-incubation of primary renal cell carcinoma (RCC) cell lines with extracellular vesicles derived from metastatic RCC cell lines enhanced cellular proliferation and invasive capacity while markedly reducing apoptotic cell proportions. Intracellular miR-224 levels were significantly upregulated in primary renal carcinoma cell lines. Extracellular miR-224 in extracellular vesicles influences patient prognosis and represents a potential prognostic biomarker in ccRCC patients (Fujii et al., 2017). Dias et al. confirmed that multiple miRNAs (miR-301a-3p, miR-200c-3p, miR-25-3p) play a crucial role in sustaining ccRCC cell proliferation by targeting PTEN and activating the PI3K/AKT pathway. The expression level of hsa-miR-301a-3p derived from EVS was again elevated in metastatic patients, highlighting its potential as a biomarker for metastatic disease (Dias et al., 2020; Alves et al., 2024). Findings by Alves et al. further support hsa-miR-200c-3p, hsa-miR-25-3p, and hsa-miR-301a-3p in extracellular vesicles as potential biomarkers for monitoring disease aggressiveness. Experiments revealed that co-inhibiting these EVs-miRNAs significantly increased PTEN expression, reduced tumour cell proliferation and migration in 2D models, and diminished spheroid size and metabolic capacity in 3D models. These EVs-miRNAs demonstrate potential as biomarkers for monitoring disease invasiveness and as therapeutic targets for ccRCC, potentially enabling more effective and personalised treatments for patients (Alves et al., 2025). Song et al. identified miR-9-5 as highly expressed in renal cancer patients’ serum, correlating with advanced TNM staging (tumour size, lymph node metastasis) and Fuhrman grade. Both *in vitro* and *in vivo* studies suggest its utility as a diagnostic biomarker and treatment response monitor (Song et al., 2020). Furthermore, Xiao et al. reported upregulation of miR-149-3p and miR-424-3p alongside significant downregulation of miR-92a-1-5p in plasma EVs, suggesting their combination holds potential for RCC screening. Plasma extracellular vesicles containing hsa-miR-92a-1-5p,hsa-miR-149-3p, and hsa-miR-424-3p in plasma extracellular vesicles may serve as potential biomarkers for detecting RCC (Xiao C. T. et al., 2020).

In urinary EVs, researchers developed a nanowire-based EV enrichment technique capable of capturing over 99% of EVs and detecting approximately 2,500 miRNAs. This approach revealed a miRNA profile similar to that in serum, with urine being a more suitable sampling source due to its proximity to the kidneys (Yasui et al., 2024). Small extracellular vesicles secreting distinct miRNA combinations, including miR-126-3p + miR-449a, miR-126-3p + miR-34b-5p, miR-126-3p + miR-486-5p, miR-25-3p + miR-34b-5p,miR-21-5p + miR-34b-5p, and miR-150-5p + miR-126-3p, have been reported as diagnostic biomarkers for clear cell renal cell carcinoma (ccRCC),with sensitivities of 60.6%, 67.3%, 52.9%, 73.1%, 74%, and 61.5%, and specificities of 100%, 82.8%, 95.8%, 79.3%, 72.4%, and 82.8% respectively (Butz et al., 2016). Qin et al. demonstrated that miR-224-5p was significantly upregulated in urine EVs from RCC patients compared to healthy volunteers. Overexpression of miR-224-5p inhibited RCC cell proliferation and induced cell cycle arrest. Urinary EVs containing miR-224-5p were identified as a potential biomarker for RCC (Qin et al., 2021). Butz et al. observed significant downregulation of miR-126-3p (P = 0.004) in urinary EVs, alongside upregulation of miRNA-150-5p, suggesting potential for tumour diagnosis using these miRNAs in SRMs.Moreover, several distinct EVs-miRNA combinations (including miR-126-3p, miR-486-5p, and miR-34b-5p) not only differentiated general ccRCC patients and SRMs from healthy participants but also distinguished benign tumour patients from ccRCC patients. These data are highly promising and may improve future surgical management decisions for SRMs (Butz et al., 2016). Moreover, Song et al. discovered that miR-30c-5p in urinary extracellular vesicles targets heat shock protein 5 (HSPA5) and inhibits ccRCC progression, exhibiting a sensitivity of 68.57% and specificity of 100%, demonstrating significant potential as a diagnostic biomarker for ccRCC (Song et al., 2019). Crentsil et al. identified miR-205 and miR-150 in extracellular vesicles as significantly differentially expressed in 786-O cells compared to controls, suggesting their utility as ccRCC extracellular vesicle biomarkers. Results from *in vitro* models corroborated this finding, though only miR-205 achieved statistical significance (Crentsil et al., 2018). Consequently, miR-224-5p, miR-126-3p, miR-150-5p, miR-30c-5p, miR-205,miR-486-5p, and miR-34b-5p have been reported to exhibit altered expression in urinary EVs, showing promise for the early diagnosis and differentiation of RCC and SRMs (Butz et al., 2016; Crentsil et al., 2018; Song et al., 2019). Notably, miR-224-5p demonstrates novel predictive value in immunotherapy by influencing the stability of Cyclin D1 and PD-L1 (Qin et al., 2021).

Several candidate miRNAs detected in RCC have also been implicated in other renal disorders, which challenges their disease specificity. For instance, miR-9-5p has been proposed as a non-invasive biomarker for idiopathic membranous nephropathy (IMN) (Guo et al., 2022), and miR-210 has shown diagnostic relevance in IgA nephropathy (IgAN) (Zhao et al., 2022). Such evidence indicates that single miRNAs may lack sufficient specificity for RCC diagnosis. However, their clinical value remains promising, particularly when applied as part of multi-marker panels or in combination with imaging and clinicopathological features to enhance diagnostic precision.

In summary, EVs-miRNAs in serum and urine have demonstrated favourable specificity and stability, offering broad prospects for non-invasive detection, prognostic assessment, and treatment response prediction in RCC. Future large-scale prospective studies and technical standardisation are required to advance their clinical translation, thereby providing more personalised and precise management strategies for RCC patients (Table 2).

**TABLE 2:** Promising diagnostic extracellular vesicle-associated microRNAs in renal cell carcinoma.

Biomarker	Clinical application	Source	Number of cases/ Controls	Sensitivity%	Specificity%	AUC	Characterization	Quantification technique	Normalizer	REF
miR-210	Diagnostic of CCRCC	Serum	82 ccRCC patients/80 healthy controls	70.000	62.200	NA	Flow cytometry analysis and immunofluorescence	qRT-PCR	U6	Zhang et al. (2018)
miR-1233				81.000	76.000					
miR-210	Diagnostic of CCRCC	Serum	45 ccRCC patients/30 healthy controls	82.500	80.000	NA	TME; Western blot	qRT-PCR	miR-16-5p	Wang et al. (2019b)
miR-224	Diagnostic of CCRCC	Serum	108 ccRCC patients	NA	NA	0.833	TME; Immunoprecipitation and western analysis	qRT-PCR	U6	Fujii et al. (2017)
miR-9-5	Diagnostic of CCRCC	Serum	35 ccRCC patients (high expression)/31 ccRCC patients (low expression)	NA	NA	NA	Western blot	qRT-PCR	snRNAU6	Song et al. (2020)
miR-149-3p	Diagnostic of CCRCC	Serum	22 ccRCC patients/16 healthy controls	75.000	77.300	0.719	TEM; NTA	qRT-PCR	miR-16-5p	Xiao et al. (2020a)
miR-424-3p				75.000	72.700	0.773				
miR-92a-1-5p				87.500	81.800	0.832				
hsa-miR-200c-3p	Diagnostic of CCRCC	2D and 3D cell culture	NA	NA	NA	NA	TEM	qRT-PCR	GAPDH	Alves et al. (2024), Alves et al. (2025)
hsa-miR-25-3p										
has-301a-3p										
miR-224-5p	Diagnostic of CCRCC	urine	NA	NA	NA	NA	TE,; NTA; Western blot	qRT-PCR	miR-16; RNU48	Qin et al. (2021)
miR-30c-5p	Diagnostic biomarker of early-stage ccRCC	urine	70 early-stage (T1aN0M0) ccRCC patients/30 early-stage prostate cancer (T1N0M0) patients/30 early-stage bladder cancer (T1N0M0) patients/30 hralthy controls	68.57	100	0.819	TEM; NTA	qRT-PCR	Not Specified	Song et al. (2019)
Combination of miR-126-3p-miR-449a	Diagnostic of CCRCC	Serum	81 ccRCC patients/24 patients with benign lesions/33 healthy controls	60.6	100	0.82	TEM	qRT-PCR	miR-16-5p + miR-106a-5p	Butz et al. (2016)
Combination of miR-126-3p-miR-34b-5p				67.3	82.8	0.8				
Combination of miR-126-3p-miR-486-5p				52.9	95.8	0.79				
Combination of miR-25-3p-miR-34b-5p				73.1	79.3	0.76				
Combination of miR-21-5p-miR-34b-5p				74	72.4	0.76				
Combination of miR-150-5p-miR-126-3p				61.5	82.8	0.76				

To ensure methodological consistency and comparability of results, only studies assessing miRNA, expression levels using qPCR, were included. This criterion was based on (1) higher sensitivity and specificity of qPCR, for low-abundance miRNAs, particularly in serum/plasma; (2) its wide use and validation in biomarker studies; (3) avoidance of bias from mixed detection methods. Certain miRNAs (e.g., miR-9-5p, miR-210) and their extracellular vesicle origins have also been reported in other kidney diseases, implying that their specificity as single RCC, biomarkers may be limited.

## 5 The potential of EVs-miRNA in the treatment of RCC

With ongoing innovations in treatment strategies, the clinical management of RCC, particularly metastatic renal cell carcinoma (mRCC), is progressively shifting towards individualisation. However, the lack of stable therapeutic predictive biomarkers, coupled with significant tumour heterogeneity, substantially increases uncertainty regarding treatment response. In recent years, the role of miRNAs within EVs in regulating RCC biological behaviour and guiding therapy has garnered considerable attention, offering novel avenues for clinical intervention.

Indeed, for stage I tumours confined to the kidney, the five-year survival rate exceeds 90%. However, this rate drops to approximately 72.5% in stages II/III. More concerning is that around 30% of RCC patients are already at stage IV at diagnosis, with a five-year survival rate of merely 12% (Padala et al., 2020). RCC exhibits widespread resistance to conventional chemotherapy and radiotherapy, with radical or partial nephrectomy remaining the standard treatment approach (Yang and Liao, 2018). However, radical surgery may lead to renal insufficiency, increased cardiovascular event risk, and elevated mortality (Alam et al., 2019). Surgical approach must be balanced against factors including tumour stage, size, and grade (Padala et al., 2020; Marchioni et al., 2021; Spadaccino et al., 2021). Despite aggressive treatment, approximately 20%–50% of patients will progress to advanced disease (Padala et al., 2020).

In recent years, combination therapy with ICIs and anti-angiogenic TKIs has significantly improved treatment response in mRCC (Borchiellini and Maillet, 2022). Nevertheless, therapeutic efficacy remains highly variable, potentially constrained by the absence of a unified molecular classification, intratumoural heterogeneity, and differences between RCC subtypes. Currently, the IMDC risk scoring model is the only prospectively validated prognostic indicator for mRCC (Dudani et al., 2020). Concurrently, a substantial proportion of patients develop primary or acquired resistance to targeted therapies (Makhov et al., 2018). Consequently, identifying novel, highly effective, and predictable molecular biomarkers is imperative.

EVs-miRNA, as key information molecules secreted by tumour cells and released into body fluids, participate in regulating multiple tumour biological processes, particularly in remodelling the tumour microenvironment (TME) (D'Souza-Schorey and Clancy, 2012). EVs modulate the state of local fibroblasts, macrophages, and vascular endothelial cells, inducing their transformation into pro-tumour subtypes that support tumour progression (Kosaka et al., 2016; Madeo et al., 2018; Ringuette Goulet et al., 2018). Furthermore, tumour derived EVs exert remote effects, inducing the formation of ‘pre-metastatic niches’ in target organs, enhancing vascular permeability, recruiting mesenchymal stem cells, and reprogramming the local matrix (Hoshino et al., 2015; Zhang L. et al., 2015). Conversely, EVs released by stromal cells can influence tumour cell behaviour, either enhancing their invasive capacity or inducing a dormant state (Roccaro et al., 2013; Ono et al., 2014). EVs also transmit drug resistance signals between primary and metastatic sites, participating in the establishment and maintenance of resistance (Qu et al., 2016; Lobb et al., 2017).

Specific studies indicate that elevated serum or plasma expression of miR-1233, miR-221, and miR-210 correlates significantly with RCC-specific mortality risk (Wulfken et al., 2011; Dias et al., 2017),with miR-1233 further proposed as a potential therapeutic target. Yoshino et al. discovered that EVs-miR-1 significantly inhibits RCC cell proliferation, migration, and invasion. Its downregulation in RCC tissues correlates with reduced patient survival, suggesting therapeutic potential (Yoshino et al., 2022). Furthermore, upregulation of HIF-1α expression in TAMs induces high expression of miR-193a-5p, which is transported to RCC cells via extracellular vesicles. This miRNA inhibits TIMP2 expression by targeting its 3′-UTR, thereby enhancing angiogenesis and tumour invasiveness. Notably, suppressing miR-193a-5p in TAM-derived extracellular vesicles significantly slows RCC progression and metastasis, offering a novel therapeutic direction targeting TAM-associated miRNAs (Liu et al., 2022). Regarding RCC resistance mechanisms, studies reveal that resistant RCC cells secrete EVs carrying long non-coding RNAs (lncRNAs). These lncRNAs competitively bind miR-34 and miR-449, activating MET and AXL pathways to mediate distant dissemination of sorafenib resistance (Qu et al., 2016). He et al. further demonstrated that tumour-derived extracellular vesicles promote sorafenib resistance by transporting miR-31-5p. This miRNA binds to the 3′-UTR of the MLH1 gene, suppressing its expression and inducing the formation of a resistant phenotype (Yu et al., 2015). Similar studies revealed that miR-31-5p enriched in extracellular vesicles within ccRCC models directly targets the DNA mismatch repair gene MLH1, acting as a key factor in resistance signalling. This suggests miR-31-5p and its target gene MLH1 may serve as important biomarkers for predicting sorafenib resistance and therapeutic targets (He et al., 2020). Furthermore, Song et al. reported that elevated RAB27A expression promotes miR-127-3p secretion by RCC cells. This miRNA, delivered via extracellular vesicles, enhances MYCN expression levels, thereby increasing tumour invasiveness. Consequently, engineered disruption of miR-127-3p′s extracellular vesicle transport holds promise as an effective therapeutic intervention strategy for metastatic RCC (Song et al., 2024).

In summary, the role of EVs-miRNAs in regulating therapeutic response to RCC is becoming increasingly evident. Their involvement in processes such as angiogenesis, immune regulation, and drug resistance signalling confers significant potential as predictive and therapeutic biomarkers, offering new avenues for achieving precision treatment in RCC.

## 6 Summary

MicroRNAs (miRNAs) derived from extracellular vesicles (EVs) play a pivotal regulatory role in multiple aspects of renal cell carcinoma (RCC), including its initiation and progression, immune modulation, and the development of drug resistance. Extensive research confirms that miR-210, miR-1233, miR-224, miR-301a-3p, and miR-31-5p are significantly enriched in RCC-associated EVs, exhibiting strong correlations with tumour staging, metastatic potential, and therapeutic response. These miRNAs regulate tumour cell proliferation, migration, and immune evasion by participating in signalling pathways such as PI3K/AKT, MET/AXL, and Wnt/β-catenin. Furthermore, they influence tumour-associated fibroblasts, macrophages, and vascular endothelial cells through extracellular vesicle-mediated intercellular communication, thereby shaping the tumour microenvironment. Moreover, EVs-miRNA persist stably in bodily fluids such as blood and urine, exhibiting excellent reproducibility and detection reliability, making them highly promising non-invasive biomarkers in the liquid biopsy field.

Nevertheless, current research faces several challenges. Firstly, standardisation of EVs extraction, identification, and miRNA analysis methods remains elusive, compromising the comparability and reproducibility of research outcomes. Secondly, the inherent high heterogeneity of RCC, with miRNA expression across distinct subtypes, different patients, and even between distinct lesions within the same patient, limits the establishment of uniform biomarkers. Furthermore, many reports remain at the correlation level, with insufficient in-depth investigation into the mechanisms of action of EVs-miRNA, particularly regarding their roles in tumour immune regulation and drug resistance pathways.

Future research should prioritise multicentre, large-scale, prospective clinical studies to systematically evaluate the accuracy and clinical utility of candidate EVs-miRNA in RCC diagnosis, prognosis, and treatment response prediction. Integrating multi-omics data—including transcriptomics, proteomics, and metabolomics—holds promise for enhancing biomarker screening precision. Furthermore, artificially synthesised or engineered extracellular vesicles offer technical feasibility for targeted miRNA delivery, potentially emerging as novel therapeutic strategies against drug resistant and metastatic RCC. Combining artificial intelligence to construct multi-factor models could further elevate the clinical utility of EVs-miRNA in personalised management.

In summary, EVs-miRNA, as a stable, specific, and reproducible molecular biomarker, is progressively emerging as a crucial breakthrough for precision diagnosis and treatment of RCC. With ongoing technological advancements and deepening mechanistic research, its clinical translational application in renal cancer holds considerable promise.

## References

[B1] Aguilar-Hernandez M. M. Rincon Camacho J. C. Galicia Garcia G. (2021). Extracellular vesicles and their associated miRNAs as potential prognostic biomarkers in chronic lymphocytic leukemia. Curr. Oncol. Rep. 23 (6), 66. 10.1007/s11912-021-01058-2 33855607

[B2] Alam R. Patel H. D. Osumah T. Srivastava A. Gorin M. A. Johnson M. H. (2019). Comparative effectiveness of management options for patients with small renal masses: a prospective cohort study. BJU Int. 123 (1), 42–50. 10.1111/bju.14490 30019484 PMC6301094

[B3] Alves Â. Medeiros R. Teixeira A. L. Dias F. (2024). Decoding PTEN regulation in clear cell renal cell carcinoma: pathway for biomarker discovery and therapeutic insights. Biochim. Biophys. Acta Rev. Cancer 1879 (5), 189165. 10.1016/j.bbcan.2024.189165 39117092

[B4] Alves Â. Ferreira M. Eiras M. Lima L. Medeiros R. Teixeira A. L. (2025). Exosome-derived hsa-miR-200c-3p, hsa-miR-25-3p and hsa-miR-301a-3p as potential biomarkers and therapeutic targets for restoration of PTEN expression in clear cell renal cell carcinoma. Int. J. Biol. Macromol. 302, 140607. 10.1016/j.ijbiomac.2025.140607 39900161

[B5] An H. J. Song D. H. Koh H. M. Ko G. H. Lee J. H. Kim D. C. (2019). RAB27A is an independent prognostic factor in clear cell renal cell carcinoma. Biomark. Med. 13 (4), 239–247. 10.2217/bmm-2018-0336 30661368

[B7] Borchiellini D. Maillet D. (2022). Clinical activity of immunotherapy-based combination first-line therapies for metastatic renal cell carcinoma: the right treatment for the right patient. Bull. Cancer 109 (2s), 2s4–2s18. 10.1016/s0007-4551(22)00234-x 35760470

[B8] Boutilier A. J. Elsawa S. F. (2021). Macrophage polarization states in the tumor microenvironment. Int. J. Mol. Sci. 22 (13), 6995. 10.3390/ijms22136995 34209703 PMC8268869

[B9] Butz H. Nofech-Mozes R. Ding Q. Khella H. W. Z. Szabó P. M. Jewett M. (2016). Exosomal MicroRNAs are diagnostic biomarkers and can mediate cell-cell communication in renal cell carcinoma. Eur. Urol. Focus 2 (2), 210–218. 10.1016/j.euf.2015.11.006 28723537

[B10] Chen X. Liang H. Zhang J. Zen K. Zhang C. Y. (2012). Secreted microRNAs: a new form of intercellular communication. Trends Cell Biol. 22 (3), 125–132. 10.1016/j.tcb.2011.12.001 22260888

[B11] Chen X. W. Yu T. J. Zhang J. Li Y. Chen H. L. Yang G. F. (2017). CYP4A in tumor-associated macrophages promotes pre-metastatic niche formation and metastasis. Oncogene 36 (35), 5045–5057. 10.1038/onc.2017.118 28481877 PMC5582214

[B12] Chen Y. Y. Hu H. H. Wang Y. N. Liu J. R. Liu H. J. Liu J. L. (2020). Metabolomics in renal cell carcinoma: from biomarker identification to pathomechanism insights. Arch. Biochem. Biophys. 695, 108623. 10.1016/j.abb.2020.108623 33039388

[B13] Chen Y. W. Wang L. Panian J. Dhanji S. Derweesh I. Rose B. (2023). Treatment landscape of renal cell carcinoma. Curr. Treat. Options Oncol. 24 (12), 1889–1916. 10.1007/s11864-023-01161-5 38153686 PMC10781877

[B14] Chow T. F. Youssef Y. M. Lianidou E. Romaschin A. D. Honey R. J. Stewart R. (2010). Differential expression profiling of microRNAs and their potential involvement in renal cell carcinoma pathogenesis. Clin. Biochem. 43 (1-2), 150–158. 10.1016/j.clinbiochem.2009.07.020 19646430

[B15] Clayton A. Turkes A. Navabi H. Mason M. D. Tabi Z. (2005). Induction of heat shock proteins in B-cell exosomes. J. Cell Sci. 118 (Pt 16), 3631–3638. 10.1242/jcs.02494 16046478

[B16] Crentsil V. C. Liu H. Sellitti D. F. (2018). Comparison of exosomal microRNAs secreted by 786-O clear cell renal carcinoma cells and HK-2 proximal tubule-derived cells in culture identifies microRNA-205 as a potential biomarker of clear cell renal carcinoma. Oncol. Lett. 16 (1), 1285–1290. 10.3892/ol.2018.8751 30061948 PMC6063036

[B17] D'Souza-Schorey C. Clancy J. W. (2012). Tumor-derived microvesicles: shedding light on novel microenvironment modulators and prospective cancer biomarkers. Genes Dev. 26 (12), 1287–1299. 10.1101/gad.192351.112 22713869 PMC3387656

[B18] De Palma G. Sallustio F. Curci C. Galleggiante V. Rutigliano M. Serino G. (2016). The three-gene signature in urinary extracellular vesicles from patients with clear cell renal cell carcinoma. J. Cancer 7 (14), 1960–1967. 10.7150/jca.16123 27877211 PMC5118659

[B19] Delcuratolo M. D. Tucci M. Turco F. Di Stefano R. F. Ungaro A. Audisio M. (2023). Therapeutic sequencing in advanced renal cell carcinoma: how to choose considering clinical and biological factors. Crit. Rev. Oncol. Hematol. 181, 103881. 10.1016/j.critrevonc.2022.103881 36427772

[B20] Dias F. Teixeira A. L. Ferreira M. Adem B. Bastos N. Vieira J. (2017). Plasmatic miR-210, miR-221 and miR-1233 profile: potential liquid biopsies candidates for renal cell carcinoma. Oncotarget 8 (61), 103315–103326. 10.18632/oncotarget.21733 29262564 PMC5732730

[B21] Dias F. Teixeira A. L. Nogueira I. Morais M. Maia J. Bodo C. (2020). Extracellular vesicles enriched in hsa-miR-301a-3p and hsa-miR-1293 dynamics in clear cell renal cell carcinoma patients: potential biomarkers of metastatic disease. Cancers (Basel) 12 (6), 1450. 10.3390/cancers12061450 32498409 PMC7352268

[B22] Ding M. Zhao X. Chen X. Diao W. Kan Y. Cao W. (2022). Cancer-associated fibroblasts promote the stemness and progression of renal cell carcinoma *via* exosomal miR-181d-5p. Cell Death Discov. 8 (1), 439. 10.1038/s41420-022-01219-7 36319622 PMC9626570

[B23] Doncheva A. I. Romero S. Ramirez-Garrastacho M. Lee S. Kolnes K. J. Tangen D. S. (2022). Extracellular vesicles and microRNAs are altered in response to exercise, insulin sensitivity and overweight. Acta Physiol. (Oxf) 236 (4), e13862. 10.1111/apha.13862 36377504 PMC9788120

[B24] Dudani S. Savard M. F. Heng D. Y. C. (2020). An update on predictive biomarkers in metastatic renal cell carcinoma. Eur. Urol. Focus 6 (1), 34–36. 10.1016/j.euf.2019.04.004 31010693

[B25] Fabbri M. (2012). TLRs as miRNA receptors. Cancer Res. 72 (24), 6333–6337. 10.1158/0008-5472.Can-12-3229 23222301

[B26] Feng J. Xu B. Dai C. Wang Y. Xie G. Yang W. (2021). Macrophage-derived exosomal miR-342-3p promotes the progression of renal cell carcinoma through the NEDD4L/CEP55 axis. Oncol. Res. 29 (5), 331–349. 10.32604/or.2022.03554 37305161 PMC10208006

[B27] Filella X. Foj L. (2017). miRNAs as novel biomarkers in the management of prostate cancer. Clin. Chem. Lab. Med. 55 (5), 715–736. 10.1515/cclm-2015-1073 26751899

[B28] Fujii N. Hirata H. Ueno K. Mori J. Oka S. Shimizu K. (2017). Extracellular miR-224 as a prognostic marker for clear cell renal cell carcinoma. Oncotarget 8 (66), 109877–109888. 10.18632/oncotarget.22436 29299115 PMC5746350

[B29] Fusco C. De Rosa G. Spatocco I. Vitiello E. Procaccini C. Frigè C. (2024). Extracellular vesicles as human therapeutics: a scoping review of the literature. J. Extracell. Vesicles 13 (5), e12433. 10.1002/jev2.12433 38738585 PMC11089593

[B30] Genova C. Marconi S. Chiorino G. Guana F. Ostano P. Santamaria S. (2024). Extracellular vesicles miR-574-5p and miR-181a-5p as prognostic markers in NSCLC patients treated with nivolumab. Clin. Exp. Med. 24 (1), 182. 10.1007/s10238-024-01427-8 39105937 PMC11303437

[B31] Goldie B. J. Dun M. D. Lin M. Smith N. D. Verrills N. M. Dayas C. V. (2014). Activity-associated miRNA are packaged in Map1b-enriched exosomes released from depolarized neurons. Nucleic Acids Res. 42 (14), 9195–9208. 10.1093/nar/gku594 25053844 PMC4132720

[B32] Grange C. Tapparo M. Collino F. Vitillo L. Damasco C. Deregibus M. C. (2011). Microvesicles released from human renal cancer stem cells stimulate angiogenesis and formation of lung premetastatic niche. Cancer Res. 71 (15), 5346–5356. 10.1158/0008-5472.Can-11-0241 21670082

[B33] Grünwald V. Hadschik B. Klümper N. Herrmann K. (2024). Kick-starting molecular theranostics in renal cell carcinoma. J. Nucl. Med. 65 (5), 744–745. 10.2967/jnumed.124.267618 38692688

[B34] Guduric-Fuchs J. O'Connor A. Camp B. O'Neill C. L. Medina R. J. Simpson D. A. (2012). Selective extracellular vesicle-mediated export of an overlapping set of microRNAs from multiple cell types. BMC Genomics 13, 357. 10.1186/1471-2164-13-357 22849433 PMC3532190

[B35] Guil S. Esteller M. (2009). DNA methylomes, histone codes and miRNAs: tying it all together. Int. J. Biochem. Cell Biol. 41 (1), 87–95. 10.1016/j.biocel.2008.09.005 18834952

[B36] Guo S. Hao H. Li S. Zhang L. Li R. (2022). Differential expression of urinary exosomal miRNA in idiopathic membranous nephropathy and evaluation of its diagnostic value. Tohoku J. Exp. Med. 256 (4), 327–336. 10.1620/tjem.2022.J002 35296567

[B37] He J. He J. Min L. He Y. Guan H. Wang J. (2020). Extracellular vesicles transmitted miR-31-5p promotes sorafenib resistance by targeting MLH1 in renal cell carcinoma. Int. J. Cancer 146 (4), 1052–1063. 10.1002/ijc.32543 31259424

[B38] Hill M. Tran N. (2021). miRNA interplay: mechanisms and consequences in cancer. Dis. Model Mech. 14 (4), dmm047662. 10.1242/dmm.047662 33973623 PMC8077553

[B39] Hoshino A. Costa-Silva B. Shen T. L. Rodrigues G. Hashimoto A. Tesic Mark M. (2015). Tumour exosome integrins determine organotropic metastasis. Nature 527 (7578), 329–335. 10.1038/nature15756 26524530 PMC4788391

[B40] Hou Y. Fan L. Li H. (2021). Oncogenic miR-27a delivered by exosomes binds to SFRP1 and promotes angiogenesis in renal clear cell carcinoma. Mol. Ther. Nucleic Acids 24, 92–103. 10.1016/j.omtn.2020.11.019 33738141 PMC7941030

[B41] Huang D. Chen J. Hu D. Xie F. Yang T. Li Z. (2021). Advances in biological function and clinical application of small extracellular vesicle membrane proteins. Front. Oncol. 11, 675940. 10.3389/fonc.2021.675940 34094979 PMC8172959

[B42] Iavello A. Frech V. S. Gai C. Deregibus M. C. Quesenberry P. J. Camussi G. (2016). Role of Alix in miRNA packaging during extracellular vesicle biogenesis. Int. J. Mol. Med. 37 (4), 958–966. 10.3892/ijmm.2016.2488 26935291 PMC4790646

[B44] Kadomoto S. Izumi K. Hiratsuka K. Nakano T. Naito R. Makino T. (2019). Tumor-associated macrophages induce migration of renal cell carcinoma cells *via* activation of the CCL20-CCR6 axis. Cancers (Basel) 12 (1), 89. 10.3390/cancers12010089 31905918 PMC7017081

[B45] Kalluri R. LeBleu V. S. (2020). The biology, function, and biomedical applications of exosomes. Science 367 (6478), eaau6977. 10.1126/science.aau6977 32029601 PMC7717626

[B46] Kita S. Shimomura I. (2022). Extracellular vesicles as an endocrine mechanism connecting distant cells. Mol. Cells 45 (11), 771–780. 10.14348/molcells.2022.0110 36380729 PMC9676990

[B47] Kogure A. Kosaka N. Ochiya T. (2019). Cross-talk between cancer cells and their neighbors *via* miRNA in extracellular vesicles: an emerging player in cancer metastasis. J. Biomed. Sci. 26 (1), 7. 10.1186/s12929-019-0500-6 30634952 PMC6330499

[B48] Komohara Y. Hasita H. Ohnishi K. Fujiwara Y. Suzu S. Eto M. (2011). Macrophage infiltration and its prognostic relevance in clear cell renal cell carcinoma. Cancer Sci. 102 (7), 1424–1431. 10.1111/j.1349-7006.2011.01945.x 21453387

[B49] Kosaka N. Iguchi H. Yoshioka Y. Takeshita F. Matsuki Y. Ochiya T. (2010). Secretory mechanisms and intercellular transfer of microRNAs in living cells. J. Biol. Chem. 285 (23), 17442–17452. 10.1074/jbc.M110.107821 20353945 PMC2878508

[B50] Kosaka N. Yoshioka Y. Fujita Y. Ochiya T. (2016). Versatile roles of extracellular vesicles in cancer. J. Clin. Invest 126 (4), 1163–1172. 10.1172/jci81130 26974161 PMC4811151

[B51] Kumar M. A. Baba S. K. Sadida H. Q. Marzooqi S. A. Jerobin J. Altemani F. H. (2024). Extracellular vesicles as tools and targets in therapy for diseases. Signal Transduct. Target Ther. 9 (1), 27. 10.1038/s41392-024-01735-1 38311623 PMC10838959

[B52] Kural S. Jain G. Agarwal S. Das P. Kumar L. (2024). Urinary extracellular vesicles-encapsulated miRNA signatures: a new paradigm for urinary bladder cancer diagnosis and classification. Urol. Oncol. 42 (7), 179–190. 10.1016/j.urolonc.2024.03.006 38594151

[B53] Larios J. Mercier V. Roux A. Gruenberg J. (2020). ALIX- and ESCRT-III-dependent sorting of tetraspanins to exosomes. J. Cell Biol. 219 (3), e201904113. 10.1083/jcb.201904113 32049272 PMC7054990

[B54] Li B. Cao Y. Sun M. Feng H. (2021a). Expression, regulation, and function of exosome-derived miRNAs in cancer progression and therapy. Faseb J. 35 (10), e21916. 10.1096/fj.202100294RR 34510546

[B55] Li D. Y. Lin F. F. Li G. P. Zeng F. C. (2021b). Exosomal microRNA-15a from ACHN cells aggravates clear cell renal cell carcinoma *via* the BTG2/PI3K/AKT axis. Kaohsiung J. Med. Sci. 37 (11), 973–982. 10.1002/kjm2.12428 34337864 PMC11896453

[B56] Linxweiler J. Junker K. (2020). Extracellular vesicles in urological malignancies: an update. Nat. Rev. Urol. 17 (1), 11–27. 10.1038/s41585-019-0261-8 31827264

[B57] Liu Q. Zhao E. Geng B. Gao S. Yu H. He X. (2022). Tumor-associated macrophage-derived exosomes transmitting miR-193a-5p promote the progression of renal cell carcinoma *via* TIMP2-dependent vasculogenic mimicry. Cell Death Dis. 13 (4), 382. 10.1038/s41419-022-04814-9 35443741 PMC9021253

[B58] Lobb R. J. van Amerongen R. Wiegmans A. Ham S. Larsen J. E. Möller A. (2017). Exosomes derived from mesenchymal non-small cell lung cancer cells promote chemoresistance. Int. J. Cancer 141 (3), 614–620. 10.1002/ijc.30752 28445609

[B59] Lu J. Getz G. Miska E. A. Alvarez-Saavedra E. Lamb J. Peck D. (2005). MicroRNA expression profiles classify human cancers. Nature 435 (7043), 834–838. 10.1038/nature03702 15944708

[B60] Lu C. Zhao Y. Wang J. Shi W. Dong F. Xin Y. (2021). Breast cancer cell-derived extracellular vesicles transfer miR-182-5p and promote breast carcinogenesis *via* the CMTM7/EGFR/AKT axis. Mol. Med. 27 (1), 78. 10.1186/s10020-021-00338-8 34294040 PMC8296627

[B61] Ma C. Ding R. Hao K. Du W. Xu L. Gao Q. (2023). Storage stability of blood samples for miRNAs in glycosylated extracellular vesicles. Molecules 29 (1), 103. 10.3390/molecules29010103 38202686 PMC10780163

[B62] Maacha S. Bhat A. A. Jimenez L. Raza A. Haris M. Uddin S. (2019). Extracellular vesicles-mediated intercellular communication: roles in the tumor microenvironment and anti-cancer drug resistance. Mol. Cancer 18 (1), 55. 10.1186/s12943-019-0965-7 30925923 PMC6441157

[B63] Madeo M. Colbert P. L. Vermeer D. W. Lucido C. T. Cain J. T. Vichaya E. G. (2018). Cancer exosomes induce tumor innervation. Nat. Commun. 9 (1), 4284. 10.1038/s41467-018-06640-0 30327461 PMC6191452

[B64] Maeda F. Adachi S. Natsume T. (2023). Non-destructive and efficient method for obtaining miRNA information in cells by artificial extracellular vesicles. Sci. Rep. 13 (1), 22231. 10.1038/s41598-023-48995-5 38097629 PMC10721859

[B65] Makarova J. Turchinovich A. Shkurnikov M. Tonevitsky A. (2021). Extracellular miRNAs and cell-cell communication: problems and prospects. Trends Biochem. Sci. 46 (8), 640–651. 10.1016/j.tibs.2021.01.007 33610425

[B66] Makhov P. Joshi S. Ghatalia P. Kutikov A. Uzzo R. G. Kolenko V. M. (2018). Resistance to systemic therapies in clear cell renal cell carcinoma: mechanisms and management strategies. Mol. Cancer Ther. 17 (7), 1355–1364. 10.1158/1535-7163.Mct-17-1299 29967214 PMC6034114

[B67] Marar C. Starich B. Wirtz D. (2021). Extracellular vesicles in immunomodulation and tumor progression. Nat. Immunol. 22 (5), 560–570. 10.1038/s41590-021-00899-0 33753940 PMC9389600

[B68] Marchioni M. Rivas J. G. Autran A. Socarras M. Albisinni S. Ferro M. (2021). Biomarkers for renal cell carcinoma recurrence: state of the art. Curr. Urol. Rep. 22 (6), 31. 10.1007/s11934-021-01050-0 33886004 PMC8062344

[B69] Marconi L. Dabestani S. Lam T. B. Hofmann F. Stewart F. Norrie J. (2016). Systematic review and meta-analysis of diagnostic accuracy of percutaneous renal tumour biopsy. Eur. Urol. 69 (4), 660–673. 10.1016/j.eururo.2015.07.072 26323946

[B70] Maruoka H. Tanaka T. Murakami H. Tsuchihashi H. Toji A. Nunode M. (2022). Cancer-specific miRNAs extracted from tissue-exudative extracellular vesicles in ovarian clear cell carcinoma. Int. J. Mol. Sci. 23 (24), 15715. 10.3390/ijms232415715 36555361 PMC9778693

[B71] Menck K. Sönmezer C. Worst T. S. Schulz M. Dihazi G. H. Streit F. (2017). Neutral sphingomyelinases control extracellular vesicles budding from the plasma membrane. J. Extracell. Vesicles 6 (1), 1378056. 10.1080/20013078.2017.1378056 29184623 PMC5699186

[B72] Mittelbrunn M. Gutiérrez-Vázquez C. Villarroya-Beltri C. González S. Sánchez-Cabo F. González M. (2011). Unidirectional transfer of microRNA-loaded exosomes from T cells to antigen-presenting cells. Nat. Commun. 2, 282. 10.1038/ncomms1285 21505438 PMC3104548

[B73] Motzer R. J. Jonasch E. Agarwal N. Alva A. Baine M. Beckermann K. (2022). Kidney cancer, version 3.2022, NCCN clinical practice guidelines in oncology. J. Natl. Compr. Canc Netw. 20 (1), 71–90. 10.6004/jnccn.2022.0001 34991070 PMC10191161

[B74] Munir J. Yoon J. K. Ryu S. (2020). Therapeutic miRNA-Enriched extracellular vesicles: current approaches and future prospects. Cells 9 (10), 2271. 10.3390/cells9102271 33050562 PMC7601381

[B75] Muth D. C. Powell B. H. Zhao Z. Witwer K. W. (2018). miRNAs in platelet-poor blood plasma and purified RNA are highly stable: a confirmatory study. BMC Res. Notes 11 (1), 273. 10.1186/s13104-018-3378-6 29728133 PMC5936026

[B76] Nawaz M. Camussi G. Valadi H. Nazarenko I. Ekström K. Wang X. (2014). The emerging role of extracellular vesicles as biomarkers for urogenital cancers. Nat. Rev. Urol. 11 (12), 688–701. 10.1038/nrurol.2014.301 25403245

[B77] Nishida-Aoki N. Ochiya T. (2015). Interactions between cancer cells and normal cells *via* miRNAs in extracellular vesicles. Cell Mol. Life Sci. 72 (10), 1849–1861. 10.1007/s00018-014-1811-0 25563488 PMC4412282

[B78] Ono M. Kosaka N. Tominaga N. Yoshioka Y. Takeshita F. Takahashi R. U. (2014). Exosomes from bone marrow mesenchymal stem cells contain a microRNA that promotes dormancy in metastatic breast cancer cells. Sci. Signal 7 (332), ra63. 10.1126/scisignal.2005231 24985346

[B79] Ortiz A. (2021). Extracellular vesicles in cancer progression. Semin. Cancer Biol. 76, 139–142. 10.1016/j.semcancer.2021.05.032 34090999 PMC9662685

[B80] Padala S. A. Barsouk A. Thandra K. C. Saginala K. Mohammed A. Vakiti A. (2020). Epidemiology of renal cell carcinoma. World J. Oncol. 11 (3), 79–87. 10.14740/wjon1279 32494314 PMC7239575

[B81] Pathria P. Louis T. L. Varner J. A. (2019). Targeting tumor-associated macrophages in cancer. Trends Immunol. 40 (4), 310–327. 10.1016/j.it.2019.02.003 30890304

[B82] Pollard J. W. (2004). Tumour-educated macrophages promote tumour progression and metastasis. Nat. Rev. Cancer 4 (1), 71–78. 10.1038/nrc1256 14708027

[B83] Pyonteck S. M. Akkari L. Schuhmacher A. J. Bowman R. L. Sevenich L. Quail D. F. (2013). CSF-1R inhibition alters macrophage polarization and blocks glioma progression. Nat. Med. 19 (10), 1264–1272. 10.1038/nm.3337 24056773 PMC3840724

[B84] Qin Z. Hu H. Sun W. Chen L. Jin S. Xu Q. (2021). miR-224-5p contained in urinary extracellular vesicles regulates PD-L1 expression by inhibiting cyclin D1 in renal cell carcinoma cells. Cancers (Basel) 13 (4), 618. 10.3390/cancers13040618 33557163 PMC7913995

[B85] Qu L. Ding J. Chen C. Wu Z. J. Liu B. Gao Y. (2016). Exosome-transmitted lncARSR promotes sunitinib resistance in renal cancer by acting as a competing endogenous RNA. Cancer Cell 29 (5), 653–668. 10.1016/j.ccell.2016.03.004 27117758

[B86] Rädler J. Gupta D. Zickler A. Andaloussi S. E. (2023). Exploiting the biogenesis of extracellular vesicles for bioengineering and therapeutic cargo loading. Mol. Ther. 31 (5), 1231–1250. 10.1016/j.ymthe.2023.02.013 36805147 PMC10188647

[B87] Rai A. Claridge B. Lozano J. Greening D. W. (2024). The discovery of extracellular vesicles and their emergence as a next-generation therapy. Circ. Res. 135 (1), 198–221. 10.1161/circresaha.123.323054 38900854

[B88] Raposo G. Stoorvogel W. (2013). Extracellular vesicles: exosomes, microvesicles, and friends. J. Cell Biol. 200 (4), 373–383. 10.1083/jcb.201211138 23420871 PMC3575529

[B89] Ries C. H. Cannarile M. A. Hoves S. Benz J. Wartha K. Runza V. (2014). Targeting tumor-associated macrophages with anti-CSF-1R antibody reveals a strategy for cancer therapy. Cancer Cell 25 (6), 846–859. 10.1016/j.ccr.2014.05.016 24898549

[B90] Ringuette Goulet C. Bernard G. Tremblay S. Chabaud S. Bolduc S. Pouliot F. (2018). Exosomes induce fibroblast differentiation into cancer-associated fibroblasts through TGFβ signaling. Mol. Cancer Res. 16 (7), 1196–1204. 10.1158/1541-7786.Mcr-17-0784 29636362

[B91] Roccaro A. M. Sacco A. Maiso P. Azab A. K. Tai Y. T. Reagan M. (2013). BM mesenchymal stromal cell-derived exosomes facilitate multiple myeloma progression. J. Clin. Invest 123 (4), 1542–1555. 10.1172/jci66517 23454749 PMC3613927

[B92] Romano R. Picca A. Eusebi L. H. U. Marzetti E. Calvani R. Moro L. (2021). Extracellular vesicles and pancreatic cancer: insights on the roles of miRNA, lncRNA, and protein cargos in cancer progression. Cells 10 (6), 1361. 10.3390/cells10061361 34205944 PMC8226820

[B93] Saeedi S. Nagy C. Ibrahim P. Théroux J. F. Wakid M. Fiori L. M. (2021). Neuron-derived extracellular vesicles enriched from plasma show altered size and miRNA cargo as a function of antidepressant drug response. Mol. Psychiatry 26 (12), 7417–7424. 10.1038/s41380-021-01255-2 34385599

[B94] Santangelo L. Giurato G. Cicchini C. Montaldo C. Mancone C. Tarallo R. (2016). The RNA-binding protein SYNCRIP is a component of the hepatocyte exosomal machinery controlling MicroRNA sorting. Cell Rep. 17 (3), 799–808. 10.1016/j.celrep.2016.09.031 27732855

[B95] Schnetz M. Meier J. K. Rehwald C. Mertens C. Urbschat A. Tomat E. (2020). The disturbed iron phenotype of tumor cells and macrophages in renal cell carcinoma influences tumor growth. Cancers (Basel) 12 (3), 530. 10.3390/cancers12030530 32106629 PMC7139531

[B96] Selvaskandan H. Pawluczyk I. Barratt J. (2023). Clinical application of microRNAs in glomerular diseases. Nephrol. Dial. Transpl. 38 (6), 1375–1384. 10.1093/ndt/gfac230 35906877

[B97] Shurtleff M. J. Temoche-Diaz M. M. Karfilis K. V. Ri S. Schekman R. (2016). Y-box protein 1 is required to sort microRNAs into exosomes in cells and in a cell-free reaction. Elife 5, e19276. 10.7554/eLife.19276 27559612 PMC5047747

[B98] Skotland T. Sagini K. Sandvig K. Llorente A. (2020). An emerging focus on lipids in extracellular vesicles. Adv. Drug Deliv. Rev. 159, 308–321. 10.1016/j.addr.2020.03.002 32151658

[B99] Song S. Long M. Yu G. Cheng Y. Yang Q. Liu J. (2019). Urinary exosome miR-30c-5p as a biomarker of clear cell renal cell carcinoma that inhibits progression by targeting HSPA5. J. Cell Mol. Med. 23 (10), 6755–6765. 10.1111/jcmm.14553 31342628 PMC6787446

[B100] Song W. Chen Y. Zhu G. Xie H. Yang Z. Li L. (2020). Exosome-mediated miR-9-5p promotes proliferation and migration of renal cancer cells both *in vitro* and *in vivo* by targeting SOCS4. Biochem. Biophys. Res. Commun. 529 (4), 1216–1224. 10.1016/j.bbrc.2020.06.114 32819588

[B101] Song D. H. Lee J. S. Lee J. H. Kim D. C. Yang J. W. Kim M. H. (2024). Exosome-mediated secretion of miR-127-3p regulated by RAB27A accelerates metastasis in renal cell carcinoma. Cancer Cell Int. 24 (1), 153. 10.1186/s12935-024-03334-0 38685086 PMC11057152

[B102] Spadaccino F. Gigante M. Netti G. S. Rocchetti M. T. Franzin R. Gesualdo L. (2021). The ambivalent role of miRNAs in carcinogenesis: involvement in renal cell carcinoma and their clinical applications. Pharm. (Basel) 14 (4), 322. 10.3390/ph14040322 33918154 PMC8065760

[B103] Sun Z. Shi K. Yang S. Liu J. Zhou Q. Wang G. (2018). Effect of exosomal miRNA on cancer biology and clinical applications. Mol. Cancer 17 (1), 147. 10.1186/s12943-018-0897-7 30309355 PMC6182840

[B104] Sun I. O. Bae Y. U. Lee H. Kim H. Jeon J. S. Noh H. (2022). Circulating miRNAs in extracellular vesicles related to treatment response in patients with idiopathic membranous nephropathy. J. Transl. Med. 20 (1), 224. 10.1186/s12967-022-03430-7 35568952 PMC9107687

[B105] Sun Y. Zhu L. Liu P. Zhang H. Guo F. Jin X. (2023). ZDHHC2-Mediated AGK palmitoylation activates AKT-mTOR signaling to reduce sunitinib sensitivity in renal cell carcinoma. Cancer Res. 83 (12), 2034–2051. 10.1158/0008-5472.Can-22-3105 37078777 PMC10267682

[B137] Stepanovska Tanturovska B. Manaila R. Fabbro D. Huwiler A. (2023). Lipids as targets for renal cell carcinoma therapy. Int. J. Mol. Sci. 24 (4). 10.3390/ijms24043272 36834678 PMC9963825

[B106] Svensson K. J. Belting M. (2013). Role of extracellular membrane vesicles in intercellular communication of the tumour microenvironment. Biochem. Soc. Trans. 41 (1), 273–276. 10.1042/bst20120248 23356296

[B107] Tabatabai T. S. Alizadeh M. Rezakhani L. Tabatabai T. S. Ehterami A. Kloucheh S. G. (2025). Unlocking the potential of EXOs in regenerative medicine: a comprehensive review. Tissue Cell 97, 103068. 10.1016/j.tice.2025.103068 40782392

[B108] Tkach M. Théry C. (2016). Communication by extracellular vesicles: where we are and where we need to Go. Cell 164 (6), 1226–1232. 10.1016/j.cell.2016.01.043 26967288

[B109] Trajkovic K. Hsu C. Chiantia S. Rajendran L. Wenzel D. Wieland F. (2008). Ceramide triggers budding of exosome vesicles into multivesicular endosomes. Science 319 (5867), 1244–1247. 10.1126/science.1153124 18309083

[B110] Ung T. H. Madsen H. J. Hellwinkel J. E. Lencioni A. M. Graner M. W. (2014). Exosome proteomics reveals transcriptional regulator proteins with potential to mediate downstream pathways. Cancer Sci. 105 (11), 1384–1392. 10.1111/cas.12534 25220623 PMC4454399

[B111] Urabe F. Kosaka N. Ito K. Kimura T. Egawa S. Ochiya T. (2020). Extracellular vesicles as biomarkers and therapeutic targets for cancer. Am. J. Physiol. Cell Physiol. 318 (1), C29–C39. 10.1152/ajpcell.00280.2019 31693397

[B112] Valadi H. Ekström K. Bossios A. Sjöstrand M. Lee J. J. Lötvall J. O. (2007). Exosome-mediated transfer of mRNAs and microRNAs is a novel mechanism of genetic exchange between cells. Nat. Cell Biol. 9 (6), 654–659. 10.1038/ncb1596 17486113

[B113] Vidal-Correoso D. Mateo S. V. Muñoz-Morales A. M. Lucas-Ruiz F. Jover-Aguilar M. Alconchel F. (2024). Cell-specific extracellular vesicles and their miRNA cargo released into the organ preservation solution during cold ischemia storage as biomarkers for liver transplant outcomes. Transplantation 108 (10), e301–e312. 10.1097/tp.0000000000005008 38578699

[B114] Villarroya-Beltri C. Gutiérrez-Vázquez C. Sánchez-Cabo F. Pérez-Hernández D. Vázquez J. Martin-Cofreces N. (2013). Sumoylated hnRNPA2B1 controls the sorting of miRNAs into exosomes through binding to specific motifs. Nat. Commun. 4, 2980. 10.1038/ncomms3980 24356509 PMC3905700

[B115] Wang L. Yang G. Zhao D. Wang J. Bai Y. Peng Q. (2019a). CD103-positive CSC exosome promotes EMT of clear cell renal cell carcinoma: role of remote MiR-19b-3p. Mol. Cancer 18 (1), 86. 10.1186/s12943-019-0997-z 30975145 PMC6458839

[B116] Wang X. Wang T. Chen C. Wu Z. Bai P. Li S. (2019b). Serum exosomal miR-210 as a potential biomarker for clear cell renal cell carcinoma. J. Cell Biochem. 120 (2), 1492–1502. 10.1002/jcb.27347 30304555

[B117] Wang W. Jo H. Park S. Kim H. Kim S. I. Han Y. (2022). Integrated analysis of ascites and plasma extracellular vesicles identifies a miRNA-based diagnostic signature in ovarian cancer. Cancer Lett. 542, 215735. 10.1016/j.canlet.2022.215735 35569696

[B118] Wei C. Yang C. Wang S. Shi D. Zhang C. Lin X. (2019). Crosstalk between cancer cells and tumor associated macrophages is required for mesenchymal circulating tumor cell-mediated colorectal cancer metastasis. Mol. Cancer 18 (1), 64. 10.1186/s12943-019-0976-4 30927925 PMC6441214

[B119] Wulfken L. M. Moritz R. Ohlmann C. Holdenrieder S. Jung V. Becker F. (2011). MicroRNAs in renal cell carcinoma: diagnostic implications of serum miR-1233 levels. PLoS One 6 (9), e25787. 10.1371/journal.pone.0025787 21984948 PMC3184173

[B120] Xiao C. T. Lai W. J. Zhu W. A. Wang H. (2020a). MicroRNA derived from circulating exosomes as noninvasive biomarkers for diagnosing renal cell carcinoma. Onco Targets Ther. 13, 10765–10774. 10.2147/ott.S271606 33122915 PMC7591082

[B121] Xiao Y. Zhong J. Zhong B. Huang J. Jiang L. Jiang Y. (2020b). Exosomes as potential sources of biomarkers in colorectal cancer. Cancer Lett. 476, 13–22. 10.1016/j.canlet.2020.01.033 32044357

[B122] Xiong Y. Chen X. Yang X. Zhang H. Li X. Wang Z. (2023). miRNA transcriptomics analysis shows miR-483-5p and miR-503-5p targeted miRNA in extracellular vesicles from severe acute pancreatitis-associated lung injury patients. Int. Immunopharmacol. 125 (Pt A), 111075. 10.1016/j.intimp.2023.111075 37864909

[B123] Xuan Z. Chen C. Tang W. Ye S. Zheng J. Zhao Y. (2021). TKI-resistant renal cancer secretes low-level exosomal miR-549a to induce vascular permeability and angiogenesis to promote tumor metastasis. Front. Cell Dev. Biol. 9, 689947. 10.3389/fcell.2021.689947 34179017 PMC8222687

[B124] Yang C. Liao Z. (2018). Comparison of radical nephrectomy and partial nephrectomy for T1 renal cell carcinoma: a meta-analysis. Urol. Int. 101 (2), 175–183. 10.1159/000490576 30089288

[B125] Yang L. Zou X. Zou J. Zhang G. (2021). A review of recent research on the role of MicroRNAs in renal cancer. Med. Sci. Monit. 27, e930639. 10.12659/msm.930639 33963171 PMC8114846

[B126] Yasui T. Natsume A. Yanagida T. Nagashima K. Washio T. Ichikawa Y. (2024). Early cancer detection *via* Multi-microRNA profiling of urinary exosomes captured by nanowires. Anal. Chem. 96 (43), 17145–17153. 10.1021/acs.analchem.4c02488 39422334 PMC11525924

[B127] Yoshino H. Tatarano S. Tamai M. Tsuruda M. Iizasa S. Arima J. (2022). Exosomal microRNA-1 and MYO15A as a target for therapy and diagnosis in renal cell carcinoma. Biochem. Biophys. Res. Commun. 630, 71–76. 10.1016/j.bbrc.2022.09.056 36150242

[B128] Yu X. Guo G. Li X. Zhang C. Huang L. Fang D. (2015). Retrospective analysis of the efficacy and safety of sorafenib in Chinese patients with metastatic renal cell carcinoma and prognostic factors related to overall survival. Med. Baltim. 94 (34), e1361. 10.1097/md.0000000000001361 26313773 PMC4602909

[B129] Zhang J. Li S. Li L. Li M. Guo C. Yao J. (2015a). Exosome and exosomal microRNA: trafficking, sorting, and function. Genomics Proteomics Bioinforma. 13 (1), 17–24. 10.1016/j.gpb.2015.02.001 25724326 PMC4411500

[B130] Zhang L. Zhang S. Yao J. Lowery F. J. Zhang Q. Huang W. C. (2015b). Microenvironment-induced PTEN loss by exosomal microRNA primes brain metastasis outgrowth. Nature 527 (7576), 100–104. 10.1038/nature15376 26479035 PMC4819404

[B131] Zhang W. Ni M. Su Y. Wang H. Zhu S. Zhao A. (2018). MicroRNAs in serum exosomes as potential biomarkers in clear-cell renal cell carcinoma. Eur. Urol. Focus 4 (3), 412–419. 10.1016/j.euf.2016.09.007 28753793

[B132] Zhang W. Zheng X. Yu Y. Zheng L. Lan J. Wu Y. (2022a). Renal cell carcinoma-derived exosomes deliver lncARSR to induce macrophage polarization and promote tumor progression *via* STAT3 pathway. Int. J. Biol. Sci. 18 (8), 3209–3222. 10.7150/ijbs.70289 35637970 PMC9134902

[B133] Zhang Z. Hu J. Ishihara M. Sharrow A. C. Flora K. He Y. (2022b). The miRNA-21-5p payload in exosomes from M2 macrophages drives tumor cell aggression *via* PTEN/akt signaling in renal cell carcinoma. Int. J. Mol. Sci. 23 (6), 3005. 10.3390/ijms23063005 35328425 PMC8949275

[B134] Zhao S. Sun Y. Mao Q. Zhou C. Chen Y. Xue D. (2022). Exosomal miR-4639 and miR-210 in plasma and urine as biomarkers in IgA nephropathy. Nephron 146 (6), 539–552. 10.1159/000523924 35381590

[B135] Zhou W. Fong M. Y. Min Y. Somlo G. Liu L. Palomares M. R. (2014). Cancer-secreted miR-105 destroys vascular endothelial barriers to promote metastasis. Cancer Cell 25 (4), 501–515. 10.1016/j.ccr.2014.03.007 24735924 PMC4016197

[B136] Zhou W. Ke S. Q. Huang Z. Flavahan W. Fang X. Paul J. (2015). Periostin secreted by glioblastoma stem cells recruits M2 tumour-associated macrophages and promotes malignant growth. Nat. Cell Biol. 17 (2), 170–182. 10.1038/ncb3090 25580734 PMC4312504

